# Characterization of the complete chloroplast genome of *Farfugium japonicum* (Asteraceae)

**DOI:** 10.1080/23802359.2021.1881928

**Published:** 2021-03-01

**Authors:** Ye Gu, Qing Ma, Yin Lu

**Affiliations:** a Department of Gastroenterology, Key Laboratory of Clinical Cancer Pharmacology and Toxicology Research of Zhejiang Province, Affiliated Hangzhou First People's Hospital, Zhejiang University School of Medicine, Hangzhou, Zhejiang, P.R.China; b College of Biological and Environmental Engineering, Zhejiang Shuren University, Hangzhou, P.R.China

**Keywords:** *Farfugium japonicum*, chloroplast genome, phylogeny

## Abstract

The complete chloroplast genome of *Farfugium japonicum*, which belongs to tribe Senecioneae (Asteraceae) was characterized. The size of the chloroplast genome is 151,222 bp in length with a large single copy (LSC) of 83,417 bp, a small single copy (SSC) of 18,125 bp, and a pair of inverted repeats of 24,840 bp. The chloroplast genome encodes a set of 133 genes, including 88 protein-coding genes, 37 tRNA genes, and 8 rRNA genes. Phylogenomic analysis based on chloroplast genomes of 18 related species revealed that *F. japonicum* is clustered with species from *Ligularia* and rooted with the other Senecioneae genus. The chloroplast genome of *Farfugium japonicum* provides an important resource for further study of molecular evolution.

*Farfugium japonicum* is a perennial herb belonging to tribe Senecioneae in the Asteraceae family. It is mainly distributed in Southeast China, Japan, and Korea. As a shade-tolerant species with big and thick leaves, *Farfugium japonicum* has also been cultivated worldwide for ornamental purpose. Meanwhile, *Farfugium japonicum* has long been recognized as a kind of medicinal and edible plant with a wide range of pharmacological effects such as anti-tumor, anti-inflammatory, and acaricidal effects (Hatanaka et al. 2014). Various bioactive chemical components have been found in *Farfugium japonicum*, including volatile oil, terpenoids (sesquiterpenes, diterpenoids, triterpenoids), phenols, alkaloids, steroids, and fatty acids. However, few studies have reported the genetic information of *Farfugium japonicum*. In this study, we assembled the complete chloroplast genome of *Farfugium japonicum* to provide genomic and genetic information for further research.

Specimen of *Farfugium japonicum* was collected from Hangzhou Botanical Garden (30.254 N, 120.11 E), Zhejiang Province, China. Total genomic DNA of *Farfugium japonicum* was extracted from fresh leaf tissue using the CTAB method (Doyle and Doyle 1987). A specimen was deposited in the herbarium of Zhejiang Shuren University (Qing Ma, maqing90@live.cn) under the voucher number MQ20-0606. The chloroplast genome was sequenced using the Illumina Hiseq Platform (Illumina, San Diego, CA) at BGI (Shenzhen, Guangdong, China). After removal of adaptor sequences, contamination and low-quality reads, a total of 15,969,152 clean reads were obtained. Genome assembly and annotation were performed using GetOrganelle software (Jin et al. 2018) and Geneious R8 (Biomatters Ltd, Auckland, New Zealand) respectively with *Ligularia veitchiana* (GenBank accession number: NC_039385.1) as the reference (GenBank accession number: JX08669.1) (Chen et al. 2018). The positions of start and stop codons as well as boundaries between exons and introns were manually corrected wherever necessary. The annotated complete chloroplast genome of *Farfugium japonicum* was deposited to GeneBank under the accession No. MT929248.

The chloroplast genome of *Farfugium japonicum* has a typical quadripartite structure consisting of a pair of inverted repeats (IRa and IRb: 24,840 bp), a small single copy (SSC) region (18,125 bp), and a large single copy (LSC) region (83,417 bp). The total length of chloroplast genome was 151,222 bp, which encodes 133 genes including 88 protein-coding genes, 37 tRNA genes, and 8 rRNA genes. The GC contents of LSC, SSC, each IR region, and the whole genome are 35.6%, 43%, 30.8%, and 37.5%, respectively.

To validate the phylogenetic position of *Farfugium japonicum* in Senecioneae, complete chloroplast genome sequences of 12 species from Senecioneae and 6 species from Heliantheae were downloaded from GenBank and aligned with MAFFT version 7.0 (Katoh et al. 2017). Phylogenomic analysis was conducted using maximum likelihood (ML) method implemented on the CIPRES Science Gateway V.3.3 (Miller et al. 2010; Stamatakis 2014). The phylogenetic tree strongly supported a close relationship between *Farfugium japonicum* and species from *Ligularia*. *Farfugium japonicum* and the *Ligularia* species formed a monophyletic group sister to the monophyletic group consisting of species from *Pericallis* and *Senecio*. In addition, all the species from Senecioneae formed a monophyletic group sister to the species from Heliantheae with high bootstrap value (Figure 1). The revealed phylogenetic relationship is generally consistent with previous phylogenetic study of tribe Senecioneae based on nuclear ITS data (Pelser et al. 2007). This study could provide important genetic information for further study on the molecular evolution and genetic diversity of *Farfugium japonicum*.

**Figure 1.**
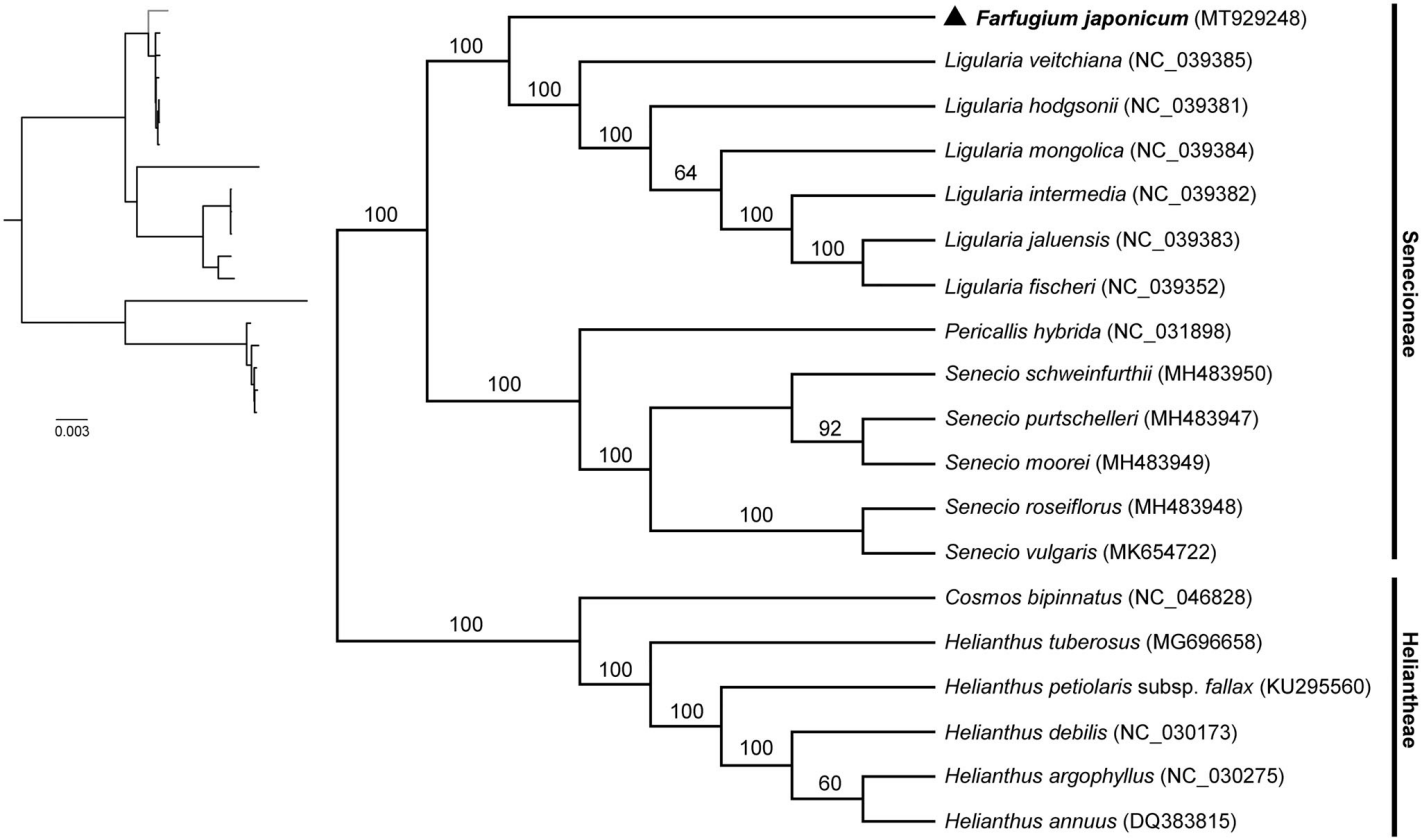
Phylogenetic tree reconstruction of *Farfugium japonicum* (marked with triangle) and other species from Senecioneae and Heliantheae using maximum likelihood (ML) based on whole chloroplast genome sequences. Relative branch lengths are indicated at the top-left corner. Numbers above the lines represent ML bootstrap values. GenBank accession numbers of all the chloroplast genome used for phylogenomic analysis are shown in the brackets.

## Data Availability

The genome sequence data that support the findings of this study are openly available in GenBank of NCBI at (https://www.ncbi.nlm.nih.gov/) under the accession no. MT929248. The associated BioProject, SRA, and Bio-Sample numbers are PRJNA658014, SRR13300173, and SAMN15856228, respectively.
